# An Unexpected Shade of Yellow

**DOI:** 10.5826/dpc.1103a52

**Published:** 2021-07-08

**Authors:** Ângela Roda, André Oliveira

**Affiliations:** 1 Department of Dermatology, Hospital de Santa Maria, Centro Hospitalar Universitário Lisboa Norte, Lisboa, Portugal; 2 Dermatology Center, Hospital CUF Descobertas, Lisboa, Portugal

**Keywords:** basal cell carcinoma, skin cancer, cholesterol, dermoscopy, histopathology

## Case Presentation

A 45-year-old woman presented with a history of a slowly growing asymptomatic solitary yellowish-red papule on the face.

**Figure 1:**
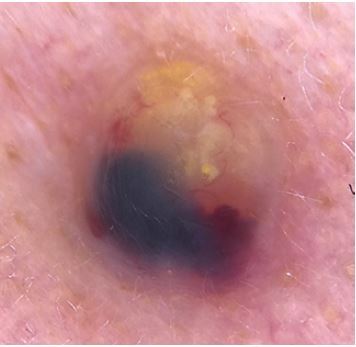
Basal cell carcinoma with cholesterol crystal deposition, dermoscopy. Yellow globules over a yellow structureless area and thin arborizing vessels are observed mainly in the upper half of the lesion. The lower portion of the lesion presents a large blue ovoid nest on the left side and a red-purple background, suggesting traumatic hemorrhage.

Dermoscopy revealed yellow globules over a yellow structureless area at the level of the upper portion of the lesion, in addition to arborizing vessels (AV). The lower half of the lesion presented a large blue ovoid nest and red-purple areas suggesting traumatic hemorrhage (Figure 1).

Histopathological examination of the skin lesion revealed a basal cell carcinoma (BCC) with cholesterol crystal deposition.

## Teaching Point

When evaluating skin tumors, color is one of the most important clues for diagnosis. In dermoscopy, the yellow color has been associated with the content of keratin, calcium, and lipids.

Yellowish structures, including milia-like cysts and yellow lobular-like structures, have already been described in BCCs [1]. Of particular interest, in our case, dermoscopic yellow structures corresponded histologically to cholesterol clefts. Cholesterol clefts have rarely been reported in cutaneous tumors other than lipid-rich tumors. However, cholesterol clefts may occur in BCC and have been associated with long-lasting disease or microtrauma [2].
